# Impact of Using GPS L2 Receiver Antenna Corrections for the Galileo E5a Frequency on Position Estimates

**DOI:** 10.3390/s20195536

**Published:** 2020-09-27

**Authors:** Andrzej Araszkiewicz, Damian Kiliszek

**Affiliations:** Faculty of Civil Engineering and Geodesy, Military University of Technology, gen. S. Kaliskiego 2, 00-908 Warsaw, Poland; damian.kiliszek@wat.edu.pl

**Keywords:** phase center variations, Galileo frequency, receiver antenna calibrations, coordinates

## Abstract

Knowledge of Global Navigation Satellite System (GNSS) antenna phase center variations plays a key role in precise positioning. Proper modeling is achieved by accessing antenna phase center corrections, which are determined in the calibration process. For most receiver antenna types, the International GNSS Service provides such corrections for two GPS and GLONASS carrier signals. In the case of Galileo, access to phase center corrections is difficult; only antennas calibrated in the anechoic chambers have available corrections for Galileo frequencies. Hence, in many of the studies, GPS-dedicated corrections are used for these Galileo frequencies. Differential analysis was conducted in this study to evaluate the impact of such change. In total, 25 stations belonging to the EUREF Permanent Network and equipped with individual calibrated antennas were the subject of this research. The results for both the absolute and relative positioning methods are clear: using GPS L2 corrections for Galileo E5a frequency causes a bias in the estimated height of almost 8 mm. For the horizontal component, a significant difference can be noticed for only one type of antenna.

## 1. Introduction

In recent years, significant development of Europe’s Global Navigation Satellite System (GNSS), Galileo, has been observed [1]. In 2011 and 2012, four In-Orbit Validation (IOV) satellites were launched, which comprised the first phase of Galileo. The next phase, Full Operational Capability (FOC), both started and launched the first two FOC satellites in 2014; however, these satellites went into the wrong orbit. Therefore, these satellites are no longer included as a part of the FOC phase of Galileo. Over the next few years, four pairs of FOC satellites were placed, and from the end of 2016, quadruple FOC satellites were placed simultaneously [2]. On 15 December, 2016, Early Operational Capability (EOC) was announced, and 12 new Galileo satellites were successfully launched between 2016 and 2018. Currently, the Galileo system has 26 satellites in space, including 22 operational, 2 with unhealth and unavailable status, and 2 in the wrong orbit. The final launch of the satellites is expected to take place in 2020, complementing and placing spare satellites before FOC is announced. At the same time, Galileo Second Generation (G2G) satellites are already being prepared that can replace the old satellites. Together with such rapid development of the Galileo system, the International GNSS Service (IGS) provides repeatedly newer and better products [3]. These works are carried out as part of the Multi-GNSS Experiment (MGEX) project, which tracks, collates and analyzes all available GNSS signals. MGEX also generates high-accuracy products, such as orbits, clocks and site coordinates [2,4,5], and it has made Galileo-based products much more accurate than they were a few years ago—even more accurate in some aspects than GPS-based products, as shown by [6]. Such accuracy is also important for multi-GNSS absolute positioning, wherein measurement accuracy increases after adding Galileo observations [1,7,8].

To estimate precisely the point position, one should model properly the transceiver system. For each GNSS signal, one should know the position of the phase center antenna (PCA) for both the transmitter (satellite) and the receiver. It is widely accepted that the PCA can be described by mean offset with respect to the antenna reference point (ARP) and its direction-dependent variation. Therefore, two definitions are used: phase center offset (PCO) and phase center variation (PCV) [9]. The IGS Antenna Working Group provides the PCO/PCV tables for satellites and receiver antennas in the standardized ANTEX format [10]. This applies to each healthy satellite and most of the ground antennas. In this format, each frequency is coded by a satellite system flag (e.g., “G” for GPS and “E” for Galileo) and a frequency number code consistent with the RINEX definitions. Initially, the IGS estimated the Galileo values for transmitting antennas as similar to GPS and GLONASS [11]. The nadir-dependent phase center variation and offsets in the z-direction for each satellite were estimated by fixing the receiver antenna phase center corrections (PCCs) to robot-based calibrations. The situation had changed by the end of January, 2018, when the IGS tables were updated (IGSMAIL-7572). Since then, the tables contain calibrations for all five carrier signals for the Galileo satellite antennas provided by the European GNSS Agency (GSA) [12]. A completely new quality for antenna modeling has arisen, since the new values are chamber-calibrated PCO/PCV and cover more space than estimated ones [13]. However, as reported by the IGS Antenna Working Group [14,15,16], these calibrations disagree with the estimated values and are not consistent with the scale of the current release of IGS (IGS14/IGb14). This translates into coordinate inconsistency on the ground. For receiver antennas, the situation is also complicated. The official IGS products contain type mean corrections for most of the commonly used antenna types, which are mean corrections that were created on the basis of many calibrations of the same type of antenna. The IGS data set is based on robot calibrations developed by the Geo++ GmbH [17], and today contains corrections for GPS (L1 = 1575.42 MHz; L2 = 1227.60 MHz) and GLONASS (G1 = 1600.995 MHz; G2 = 1248.06 MHz) frequencies only. In the ANTEX format, these signals are coded as “G01”, “G02”, “R01” and “R02” respectively. The current Geo++ capabilities already allow one to calibrate antennas for 11 GNSS frequencies [18], including Galileo. As reported by [15], the Geo++ has already delivered a set of 37 mean-type calibrations for IGS tests and the repro3 project. Additionally, other centers such as the Swiss Federal Institute of Technology in Zurich [19] are starting robot calibration for Galileo signals, but these products are not yet widely available. Another option is to use the calibrations performed in the anechoic chambers. This method is based on a generated artificial signal, not the real transmitted signals from satellites, but all of the frequencies needed are usually obtained. An example is the calibrations performed by the University of Bonn, Institute of Geodesy and Geoinformation [20]. The compatibility between chamber calibrations and robot calibrations is on a millimeter level [18], and is constantly the subject of discussion and analysis [21,22,23,24].

At present, until IGS releases new products, during the processing of Galileo observations, one has to use individual chamber calibrations. Unfortunately, not all antennas are individually calibrated, and even less have full tables that include all of the Galileo frequencies (E1 = 1575.42 MHz, E5a = 1176.45 MHz, E5b = 1207.140 MHz, E6 = 1278.75 MHz, and E5 = 1191.795 MHz, coded in the ANTEX format as “E01 “, “E05”, “E07”, ”E06”, and “E08”). This leads to a situation in which one will need to use GPS-dedicated corrections for the corresponding Galileo frequencies. Generally, GPS corrections for L1 and L2 are used for the Galileo E1 and E5a frequencies, respectively, in many studies [25]. In the case of E1, this is not a major problem, because the frequencies L1 and E1 are equal. Unfortunately, this is not the case for GPS L2 and Galileo E5a, since they differ by 51.15 MHz. As reported by [26], the PCC for E5a differs from the PCC for L2 by roughly a few millimeters. Therefore, using GPS-based PCC values for Galileo frequencies will introduce an error into the estimated values. The full operational capability of Galileo is practically being achieved already, and the expectations of including Galileo observations in many scientific and administrative works are steadily increasing. This applies also to works regarding the maintenance of the reference frame, one of the most precise and demanding products of the International Association of Geodesy (IAG). Of course, the problem of modeling PCO/PVC for Galileo antennas has a much broader meaning. It is related not only with ground antennas, but also with satellite antennas and related products such as orbits or GNSS-derived frame parameters. This paper concerns the problem faced by most ordinary GNSS users and focuses on the impact of using the GPS L2 receiver antenna PCC for the Galileo E5a frequency. Our motivation was to show how big the error in the coordinates can be with this approach. The analyses were conducted both for absolute and relative positioning, using the EUREF Permanent GNSS Network (EPN) as an example.

## 2. Methodology

To evaluate the impact of using various PCCs on the E5a signal, we prepared five solutions for absolute precise point positioning (PPP) and five for the relative double differences (DD) approach. Three solutions were prepared for Galileo and two for GPS data, respectively (Table 1). We used 30 s data collected between 30 June 2019 and 6 July 2019 (GPS week 2060) at stations belonging to EPN [27]. During this period, 198 stations provided Galileo data in the RINEX 3 format, which are subject to ongoing checks and validation by the EPN Central Bureau [28].

**Figure 1:**
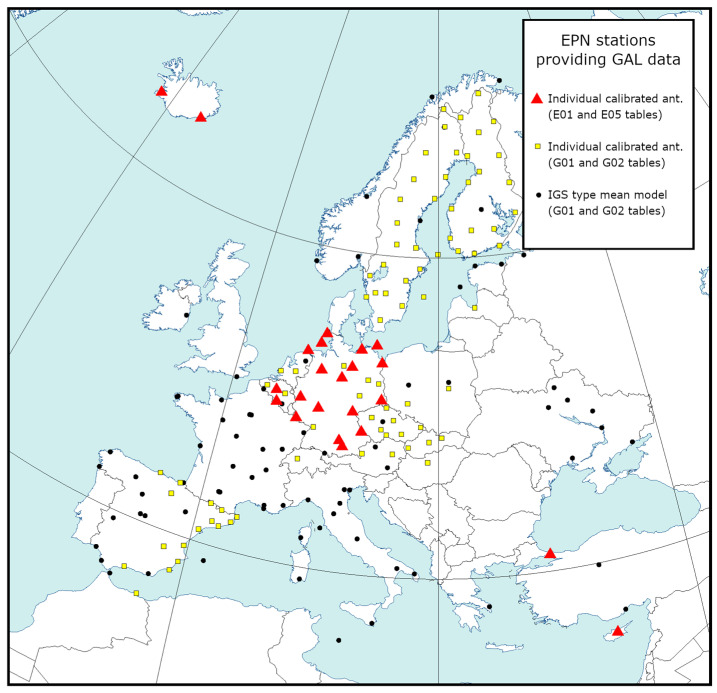
EPN stations providing the Galileo (GAL) observations between 30 June and 6 July 2019 (GPS week 2060) used in this study. NYA200NOR is outside of the presented area and is not plotted on the map.

In the standard EPN processing of model antennas, we used receiver antenna corrections from individual calibrations (epncb.atx) as a priority (robot or chamber). If this was not possible, then we used the latest IGS-type mean model release (igs14.atx) [29]. In both cases, the calibration table contains corrections for both GPS frequencies; however, in the case of Galileo, only the chamber calibrations provided us the necessary corrections for the Galileo frequencies. Unfortunately, for the analyzed period, this applied to 25 antennas only (marked as red triangles in Figure 1). In our case, all of these antennas were calibrated by the University of Bonn and were the subject of ongoing analyses. A list of the stations and mounted antennas is shown in Table 2.

The GNSS processing in the DD approach was done using the GNSS analysis software GAMIT [30], version 10.71. In this case, the analysis covered not only the selected stations (Table 3), but all of the EPN stations provided both GPS and Galileo data. The number of stations for these solutions was increased to reduce the potential impact of the different selection and process methods on the individual solutions of the 25 stations in the reference frame. The final set of 198 selected stations was divided into six clusters of a similar station density, including five tie stations per each cluster. The final number of stations per cluster ranged from 41 to 44. Observations in each cluster were processed according to the Guidelines for EPN Analysis Centres (ACs), independently for GPS and Galileo. The strategy was similar to the MUT’s (Military University of Technology, Poland) official contribution to EUREF. Together with the input of other EPN ACs, a basis was formed for determining the most accurate and up-to-date EPN station positions. This is especially important as the EPN stations support the maintenance of the European Terrestrial Reference System 89 (ETRS89) [27]. In our DD processing, an ionosphere-free combination of the L1 and L2 (E1 and E5a) carrier phases was used as the basic observations. Solid earth tidal displacements were modeled according to the International Earth Rotation and Reference Systems Service (IERS) Conventions 2010. Absolute phase center corrections were applied for the satellite antennas (igs14_2062.atx) and for the receiver antennas (details shown in Table 1). The rest of the models used are summarized in Table 3.

The processing strategy for E1, E2 and E3 was consistent at all points except one. As the receiver antenna phase center corrections for the selected 25 antennas, we used different values from the sources. In the first solution (E1), we used original Galileo-based values from the chamber calibrations. For the second solution (E2), we used GPS-based values from the same chamber calibrations. All of the remaining stations for these two solutions were processed in the same way using the original calibrations as are. Depending on the availability, the PCCs came from individual robot calibrations (performed by Geo++ GmbH mostly) or the IGS-type mean models (also based on Geo++ GmbH calibrations). In the third solution (E3), we completely skipped individual calibrations and used the IGS-type mean models [31]. Antennas at all stations, including the selected 25, were modeled with GPS-based correction values. For GPS, we produce only two solutions. The first (G1) was prepared according to EPN guidelines, and individual calibrations (both chamber and robot) were taken into account as a priority. For the second one (G2), we used only the IGS-type mean models. All five solutions were adjusted to the IGS14 framework by the list of 22 fiducial stations.

We also prepared analogous solutions for the PPP approach using GAMP software [32], but only for the investigated 25 stations. A basic PPP model was used in the analysis. Undifferenced dual-frequency codes and phase measurements were converted into an ionosphere-free linear combination [33]. In this way, we estimated the station coordinates, the clock correction of the receiver, the wet component of the tropospheric delay, and real ambiguity values. To avoid the impact of less accurate coordinates, we skipped the first epochs (1 h) in the final solution. A shortlist of used parameters for both approaches is shown in Table 3. For both approaches, daily coordinate solutions were finally prepared.

## 3. Results

In our analysis, we focused on two aspects, the primary of which concerned the impact of using GPS-based receiver antenna phase center corrections (G02) for the Galileo E5a frequency. At present, such a procedure applies to almost 90% of EPN stations providing Galileo observations. To evaluate this impact, we compared the coordinates of 25 stations that were modeled in three variants, namely E1, E2 and E3, separately for both positioning methods. It should be remembered here that antenna modeling impacts on all estimates, such as tropospheric delay or clocks. However, previously conducted studies on receiver antennas show that the greatest impact of using different PCCs is more visible in the height [23], and much less visible in the tropospheric parameters [34,35]. Therefore, in our study, we focused only on the coordinates. Typically, the daily coordinates in the PPP approach are a sample-mean of 2760 samples. In the DD approach we have one set of coordinates per day from the least squares method. Based on the daily solutions, we prepared daily coordinate differences for each station—one per day. Then, we calculated the weekly weighted averages of the differences and their standard deviations. There were typically 7 daily differences recorded. We conducted a differential analysis based on comparable solutions to eliminate software- or approach-dependent issues. We did not compare directly the results from both approaches, since the models and algorithms are completely different, and the coordinate differences would be affected by the impact of other factors as well.

**Figure 2:**
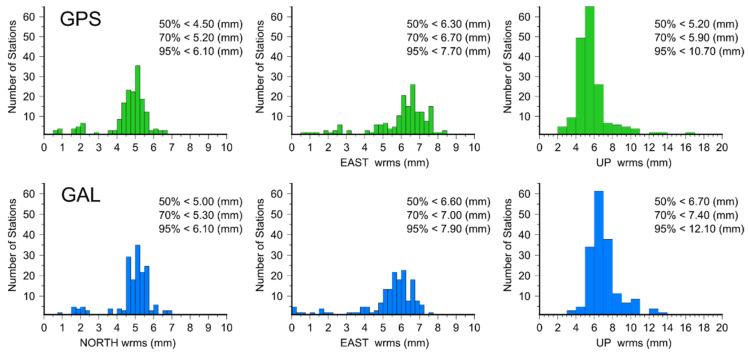
Coordinate repeatability of the GPS and Galileo solutions for the DD approach.

Second, the analysis concerned the coordinate agreement between solutions derived from GPS and Galileo data. Here, we compared the corresponding solutions: E1–G1 (according to EPN guidelines), E2–G1 (in the absence of chamber calibrations), and E3–G2 (in the absence of any individual calibrations). The general quality of the Galileo solutions has already been proven by others [1,6]. The coordinate repeatability for GPS and Galileo is almost on the same level for the EPN stations (Figure 2), despite the differences in constellation and number of observations. As both can provide different results [1], we focused only on how this agreement changed for different PCCs.

### 3.1. Differences between Galileo Solutions

**Figure 3:**
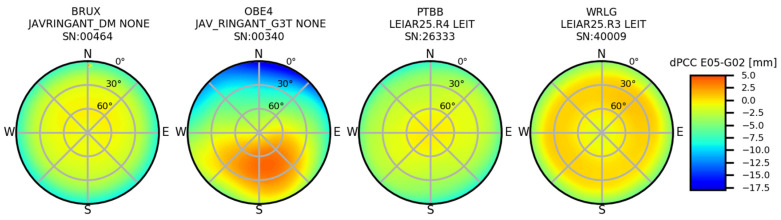
Differences in PCC (dPCC) between the E05 and G02 tables. Source: University of Bonn calibrations.

The impact of using various PCCs can be estimated initially based on the difference in the compared PCCs (dPCCs). By studying the distribution of the dPCCs, one can preliminary predict its impact height (azimuthal symmetry of dPCCs) and its horizontal component (azimuthal asymmetry of dPCCs) [36]. Such a pattern in dPCCs was already tested by [24,37] for the LEIAR25 antennas, and their results were very promising. In our case, azimuthal symmetry occurred for the majority of the antennas in the dPCCs (E05 minus G02). On this basis, one can expect that the impact of using GPS-based corrections instead of Galileo-based ones should be visible mainly in the height (Figure 3). The one exception is the JAV_RINGATN_G3T antenna, which will be discussed later.

For the horizontal component, we received, in general, consistent results for both GPS- and Galileo-based PCCs. The average difference in the coordinates for both approaches was not significant, and was 1.3 mm (north) and 0.2 mm (east) for DD and 0.6 mm (north) and −0.2 mm (east) for PPP. Therefore, one can conclude that using GPS-based receiver antenna corrections does not significantly affect the horizontal component. The one exception is the JAV_RINGANT_G3T antenna mounted at the OBE400DEU and NYA200NOR stations, for which the bias for the north component is −9.9 mm and −9.1 mm for DD and PPP, respectively. This results from the clear asymmetry in the dPCCs (Figure 3). A similar effect was visible in the work of [23] for the AOAD/M_T NONE antenna mounted at the METS00FIN station, where the high asymmetry in the dPCC impacted mostly the horizontal component effectively.

**Figure 4:**
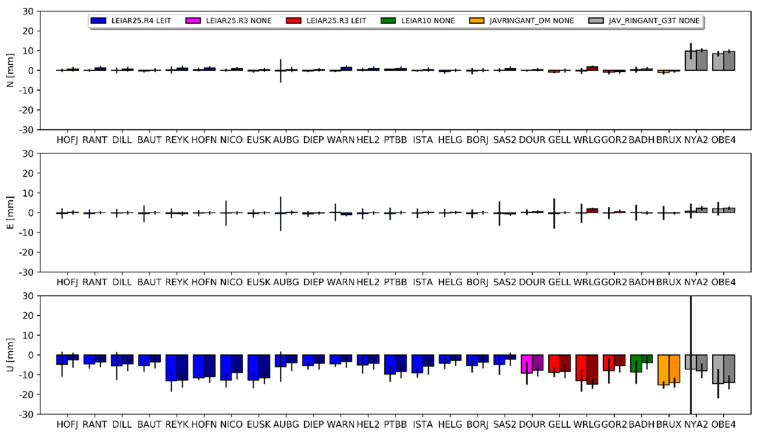
Coordinate differences between the E1 and E2 solutions and their standard deviations. Darker columns refer to the DD approach. Values are presented in Table A1 (Appendix A).

**Figure 5:**
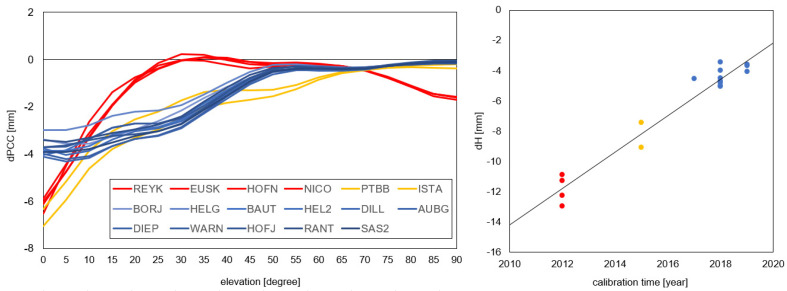
Differences in PCV between the E05 and G02 values for the LEIAR25.R4 LEIT antennas (on the left) and the resulting height differences for the DD approach (E2–E1).

The height differences varied from −2.2 mm for SAS200DEU (DD) to −15.2 mm for BRUX00BEL (PPP). The discrepancy was quite large, but the correctness of the results was confirmed by a very high consistency between both approaches (Figure 4). The mean agreement of the height differences received from DD and PPP was at the 2 mm level. However, it should also be noted here that in our study, we tried to homogenize the strategy at some critical points (e.g., cut off elevation) to minimize the discrepancies. We also observed a high discrepancy even for antennas of the same type, but we did not notice a latitude dependency here—the discrepancies came directly from the dPCC patterns (Figure 3). If we look at the dPCCs for the LEIAR25.R4 LEIT antennas, we can distinguish three groups (Figure 5). The first one contains antennas calibrated in 2012 (i.e., REYK00ISL, HOFN00ISL, NICO00CYP, and EUSK00DEU), the second group calibrated in 2015 (i.e., PTBB00DEU and ISTA00TUR), and the last group calibrated in 2017 and later. A clear dependence can be observed between the time of calibration and the dPPCs (Figure 5). The highest differences for both approaches were derived from the oldest calibrations. The mean value for both approaches was −11.9 mm. A slightly smaller difference occurred in the second group (−8.3 mm), while for the latest calibrations, the mean difference was −4.3 mm. Moreover, for the other antenna types, the oldest calibrations (before 2015) exhibited higher dPCC values, resulting in greater height differences (e.g., WRLG compared to GELL and GOR2). This may be related to the hardware modernizations of the anechoic chamber carried out in 2016, and as indicated by the University of Bonn, such modernization may affect the calibration parameters of some antenna types (Florian Zimmerman, personal communication). Unfortunately, this does not explain fully the differences between the antennas calibrated in 2012 and 2015. It is also possible that the manufacturing time of the antennas could have caused this issue, which opens up a new area of research. However, in our analysis, a higher correlation was noted for calibration time (r = 0.97) than for manufacturing time (r = 0.30).

The overall bias in height between the E1 and E2 solutions (Figure 4) was −7.0 mm for DD and −8.4 mm for PPP. The standard deviations for this difference were 4.0 mm and 3.5 mm, respectively. Similar results occurred for the E1–E3 comparison, but here the bias in height was approximately 1 mm larger (Table A2, Appendix A).

### 3.2. Differences between the GPS and Galileo Solutions

**Figure 6:**
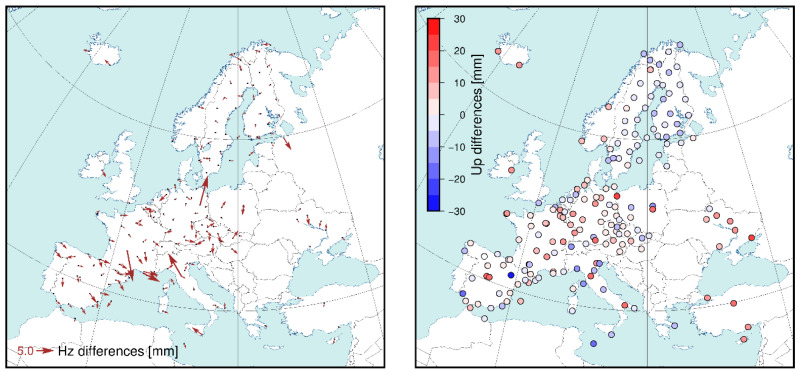
Coordinate differences between solutions E1 and G1 for the DD approach.

Before comparing solutions in the DD approach, we checked whether the G and E solutions could be directly compared. As already mentioned, we used 22 IGS stations that provide Galileo data. The mean coordinate difference at the reference stations was 0.0/0.0/1.6 mm (north/east/up), and at all stations was −0.1/−0.1/−0.2 mm (north/east/up). We did not see any visible network effect or scale issue either (Figure 6.). For individual stations, we observed a large (up to cm) difference in the horizontal component, but in spite of this, we concluded that the solutions were in the same frame and that there were no contradictions for further analysis.

**Figure 7:**
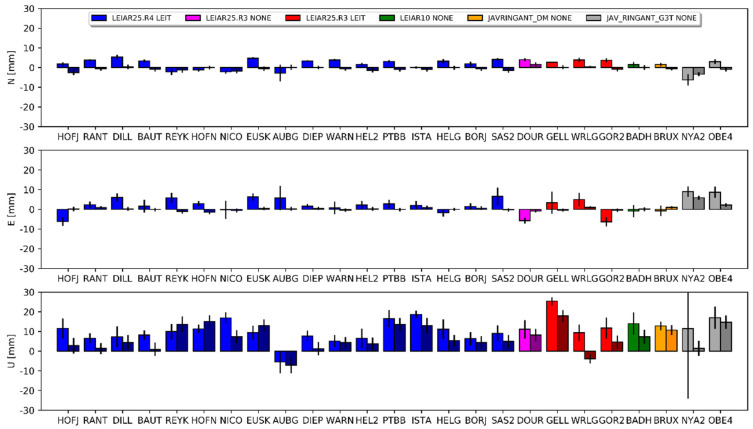
Coordinate differences between the E1 and G1 solutions and their standard deviations. Darker columns refer to the DD approach. Values are presented in Table A3.

**Figure 8:**
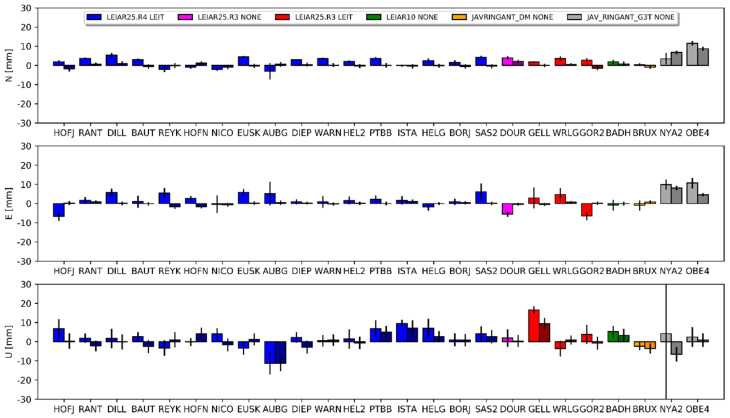
Coordinate differences between the E2 and G1 solutions and their standard deviations. Darker columns refer to the DD approach. Values are presented in Table A4.

**Figure 9:**
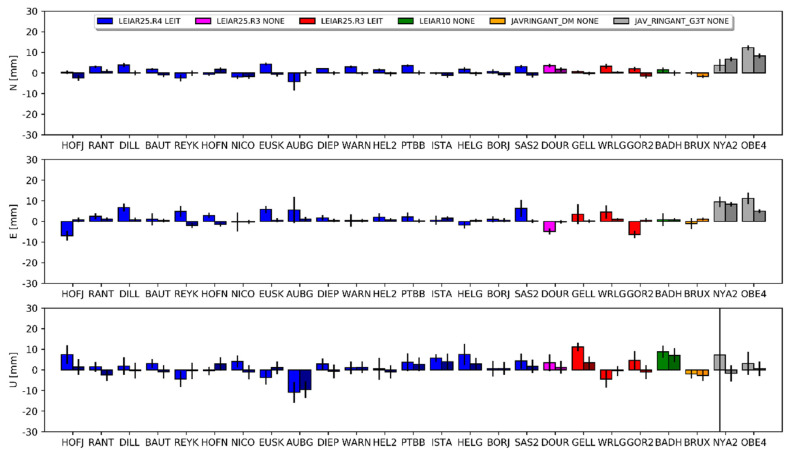
Coordinate differences between the E3 and G2 solutions and their standard deviations. Darker columns refer to the DD approach. Values are presented in Table A5.

The mean differences for the horizontal component was approximately 2 mm for the PPP approach and approximately 0.5 mm for the DD approach for all of the compared solutions (i.e., E1–G1, E2–G1 and E3–G2). The discrepancies in the estimated differences between both approaches reached a few millimeters. Detailed results for each station are presented in Figure 7, Figure 8 and Figure 9, and the corresponding values in Appendix A (Table A3, Table A4 and Table A5). The JAV_RINGANT_G3T antenna showed the biggest differences; a positive bias can be observed in both approaches in the east component, as well as a positive bias in the north component for the E2 (Figure 8) and E3 (Figure 9) solutions. Therefore, we can conclude that for one type of antenna, using proper PCCs for the E5a signal (solution E1) ensures the better compatibility of the Galileo solutions with the GPS solutions in the horizontal plane. When we used the G02 values instead of the E05 value (solutions E2 and E3), we introduced a bias of up to 1 cm. For the rest of the antennas, one can conclude that the consistency in the horizontal plane is satisfactory regardless of the used PCC values.

The situation is different for the estimated height. For the first pair (E1 and G1), a clear positive bias can be observed −6.5 mm for DD and 10.8 mm for PPP (Figure 7). Only for the AUBG station did we notice a negative (−5.5 mm and −7.1 mm) bias for both approaches. There was no visible dependency between the antenna models and the received differences. Therefore, this should be considered as a systematic error between the GPS and Galileo solutions. As shown in the work of [14], using full chamber calibrations in conjunction with the calibrations provided by the GSA for satellite antennas causes an inter-system GPS–Galileo bias. The authors indicated that this bias was present in the up component only and reached 9 mm. Our outcome confirms the results of the above work: the lower value for the DD approach (visible also in Figure 4) probably resulted from the absorption part of the common signals, which is characteristic of the regional solution [38].

Interesting here are the results for the E2 and E3 solutions. In these solutions, we skipped the E05 corrections and used G02 (individual or mean-type values) instead. The mean difference in the height for E2–G1 was 2.4 mm for PPP and 0.3 mm for DD (Figure 8). We received almost the same results for the IGS-type mean models (E3–G2), where the mean difference was 2.2 mm for PPP and 0.4 mm for DD (Figure 9). For individual stations, the differences varied from −11.2 mm to 16.6 mm. Importantly, the differences between approaches were similar for the compared pairs of solutions. We also observed a consistent effect when changing the source of G02 corrections from chamber calibrations to the IGS-type mean models between both approaches. As indicated earlier, using GPS-based correction caused a negative bias in the height (Figure 4). Here, we observed a positive inter-system GPS–Galileo bias in height (Figure 8). The overlapping of these two errors compensated for one another, as a result of which the E2 and E3 solutions were more consistent with the GPS solutions (Figure 8 and Figure 9). This has already been confirmed by the IGS [39,40] in a study of the inclusion of Galileo observations in analyses.

### 3.3. Differences between the GPS Solutions

As part of the analysis, we also compared solutions G1 and G2. This allowed us to assess the impact of differences between chamber and robot calibrations at the coordinate level. The direct comparison of Geo++ and University Bonn IGG products has already been carried out several times by others [21,41], and the authors have shown a very good agreement in L1 patterns and some systematic differences in L2 patterns. This was confirmed in the work of [42], where the authors also compared these antenna calibrations, but they focused on the estimated position. A two-fold better agreement in the estimated height was derived for the L2 signals rather than the L1 signals. In another study [22], the authors analyzed the impact of using both calibrations for six antennas. They noticed a position agreement of 2 mm in the horizontal component and 5 mm in the height. Further research into the effects of using IGS-type mean models interchangeably with various individual calibrations for the EPN network has also been conducted. In the framework of the EPN reprocessing campaign [23], it has been shown that the mean differences in all affected EPN stations were below 2 mm for the horizontal component and 4 mm for the height.

**Figure 10:**
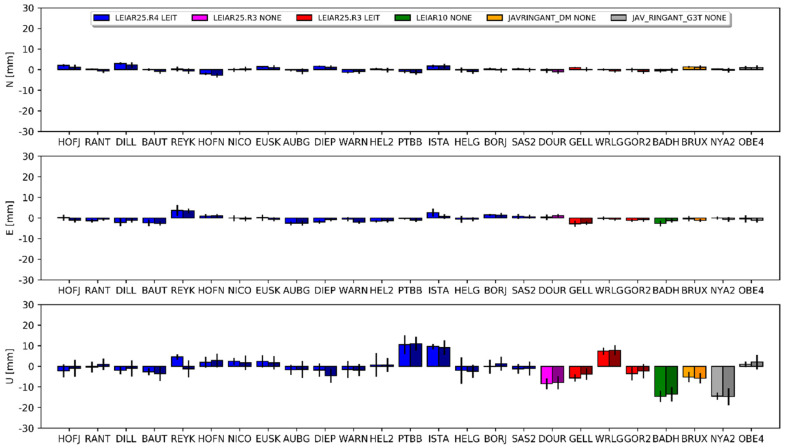
Coordinate differences between the G2 and G1 solutions and their standard deviations. Darker columns refer to the DD approach. Values are presented in Table A6.

**Figure 11:**
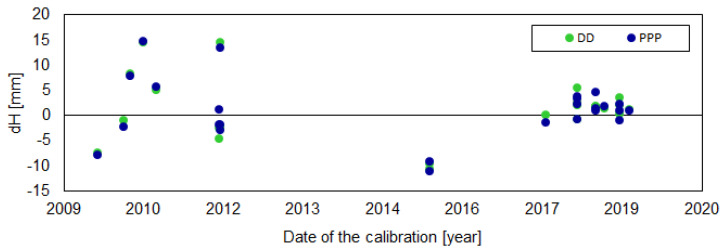
Dependence between height differences (G2–G1) and time of the calibrations.

In the current study, the differences for the horizontal component in both approaches did not exceed 4 mm (Figure 10). Again, we received very consistent results between both positioning methods. The mean differences for DD were 0.3 mm (north), 0.6 mm (east) and 1.1 mm (up), and 0.4 mm, 0.4 mm and 1.1 mm for PPP, respectively. The standard deviations of these differences for both approaches were very similar: 1.1 mm, 1.4 mm and 5.7 for DD, and 1.1 mm, 1.6 mm and 5.9 mm for PPP. The differences between both approaches for the horizontal component were within the range of −1.9 mm to 2.5 mm, with standard deviations of 0.6 mm for the north and 0.8 for the east components. For the height, the largest differences were observed for NYA200NOR, BADH00DEU and PTBB00DEU (Table A6, Appendix A), wherein it exceeded 10 mm. As one can see (Figure 9), the received differences were smaller than the differences obtained for Galileo (Section 3.1), which can only be considered significant for eight antennas. We also observed better agreement between the received coordinates for the newest calibrations, which demonstrates the growing compatibility between robot and chamber calibrations (Figure 11).

## 4. Conclusions

Our research was aimed at checking the effect of using GPS-based ground antenna phase center corrections for the Galileo E5a frequency. For many of the antennas, we did not have the full calibrations containing Galileo-specified values, and we were forced to use substitutes. In the case of the most commonly used second L-band frequencies (L2 and E5a), this is especially important, because both the frequencies and the corresponding phase center corrections are unequal and may affect the estimated values. We showed that, except for the JAV_RINGANT_G3T NONE antennas, such an approach does not cause significant positional differences in the horizontal component. However, it should be noted here that we tested only six types of antenna. Perhaps there are other models of antennas that have the same problem, but at the moment, this cannot be verified. As shown, using G02 values introduced an average bias in the height of −7.7 mm, with a standard deviation of 3.8 mm. For the latest calibrations, this bias was lower (−4.8 mm), with a standard deviation of 1.6 mm (Table 4). It can be assumed here that the stations providing Galileo observations and equipped with uncalibrated individual antennas are affected in a similar way. In our case, this applies to 172 stations. Publication of new IGS products containing receiver antenna corrections for Galileo will help this matter; however, it is very important to further calibrate the antennas for Galileo frequencies.

On the other hand, Table 5 clearly shows that using the E05 values made the Galileo solutions less consistent with the GPS solutions (E1–G1) than using G02 values (E2–G1 and E3–G2). The differences show a bias of 6.5 mm for DD and 10.8 mm for PPP approaches respectively for the E1–G1 comparison, while for E2–G1 and E3–G2 the differences are not significant. This issue was already indicated by the IGS Antenna Working Group and is related to the incompatibility between the calibrations for the satellite antennas provided by the GSA and the current IGS products, which causes an additional inter-system GPS–Galileo bias in height. However, the IGS assumes that the new release of IGS products should solve this problem. Herein, both errors compensated for one another, which resulted in a higher consistency between the GPS and Galileo solutions. However, the resulting high agreement between the GPS and Galileo solutions does not reflect the true consistency. Only when the true consistency is reflected will it be possible to assess the real agreement between the GPS and Galileo estimates.

A supplementary comparison of the chamber and robot calibrations for the GPS solutions showed good agreement between the final coordinates. The individual differences below 3 mm and mean values at the sub-millimeter level indicate that both methods were very consistent. Slightly worse results pertaining to height for individual stations applied only to calibrations carried out long ago. For the latest calibrations carried out after 2016, the mean difference was 1.7 mm, with a standard deviation of 1.6 mm, clearly showing good compatibility between the robot and chamber calibrations. Compared to the Galileo issues, this should be treated as a minor concern (Table 5).

## Figures and Tables

**Table 1:** Solution types prepared for the DD and PPP approaches. For each solution receiver, the antennas were modeled using different sources of the PCCs.

	Galileo Solutions			GPS Solutions	
	E1	E2	E3	G1	G2
PCCs	E01, E05	E01, G02	E01, G02	G01, G02	G01, G02
Source	Bonn	Bonn	IGS	Bonn	IGS

DD, double differences approach; PPP, precise point positioning; PCC, phase center corrections; IGS, IGS-type mean model (igs14.atx); Bonn, individual calibrations made by the University of Bonn (stored in epnc.atx).

**Table 2:** List of the EUREF Permanent GNSS Network (EPN) stations equipped with antennas calibrated by the University of Bonn. The calibration tables for the listed stations contain at least G01, G02, E01 and E05 corrections.

Station	Antenna Type	Serial Number	Calibration Time	Manufacturer
NYA200NOR	JAV_RINGANT_G3T NONE	342	11.11.2010	Javad, San Jose, USA
OBE400DEU	JAV_RINGANT_G3T NONE	340	14.07.2010	
BRUX00BEL	JAVRINGANT_DM NONE	464	01.02.2011	
BADH00DEU	LEIAR10 NONE	12356022	08.03.2012	Leica Geosystems AG, Heerbrugg, Switzerland
GELL00DEU	LEIAR25.R3 LEIT	10170027	25.04.2018	
GOR200DEU	LEIAR25.R3 LEIT	08500024	18.01.2019	
WRLG00DEU	LEIAR25.R3 LEIT	10240009	03.12.2010	
DOUR00BEL	LEIAR25.R3 NONE	09300021	23.08.2010	
AUBG00DEU	LEIAR25.R4 LEIT	725552	23.08.2018	
BAUT00DEU	LEIAR25.R4 LEIT	725267	25.04.2018	
BORJ00DEU	LEIAR25.R4 LEIT	726363	13.10.2017	
DIEP00DEU	LEIAR25.R4 LEIT	725557	23.08.2018	
DILL00DEU	LEIAR25.R4 LEIT	725266	23.08.2018	
EUSK00DEU	LEIAR25.R4 LEIT	725299	02.03.2012	
HEL200DEU	LEIAR25.R4 LEIT	726209	25.04.2018	
HELG00DEU	LEIAR25.R4 LEIT	726342	24.04.2018	
HOFJ00DEU	LEIAR25.R4 LEIT	10211013	18.01.2019	
HOFN00ISL	LEIAR25.R4 LEIT	725283	07.03.2012	
ISTA00TUR	LEIAR25.R4 LEIT	726339	13.10.2015	
NICO00CYP	LEIAR25.R4 LEIT	725285	07.03.2012	
PTBB00DEU	LEIAR25.R4 LEIT	726333	13.10.2015	
RANT00DEU	LEIAR25.R4 LEIT	726365	18.01.2019	
REYK00ISL	LEIAR25.R4 LEIT	725281	01.03.2012	
SAS200DEU	LEIAR25.R4 LEIT	10231013	19.03.2019	
WARN00DEU	LEIAR25.R4 LEIT	725559	15.10.2018	

**Table 3:** Processing parameters adopted for both approaches.

Approach	DD	PPP
Software	GAMIT/GLOBK 10.71	GAMP
System	GPS and Galileo separately	
Frequencies	L1/L2 for GPS and E1/E5a for Galileo	
Observations	Ionosphere-free code and phase combination	
Elevation mask	7 deg.	
Orbits	CODE MGEX	
Transmitter PCC	igs14.atx	
Receiver PCC	Depending on the solution: E1, E2, E3, G1, and G2	
Troposphere delay	VMF1 1 h ZTD and 24 h grad	GMF ZTD and grad every epoch
Clock errors	Estimated	
Ambiguities	Fixed	Float
EOP	IERS2019	
Tide displacement	IERS2010, FES2004	

CODE, Center for Orbit Determination in Europe; VMF1, Vienna Mapping Function; GMF, global mapping function; ZTD, zenith total delay.

**Table 4:** Mean differences and standard deviations in the up component between the compared solutions for the 25 analyzed stations (based on both approaches).

Calibrated Antennas	E1–E2	E1–E3	G1–G2
All (N = 25)	−7.7 ± 3.7 mm	−8.8 ± 5.3 mm	1.1 ± 5.7 mm
Before 2016 (N = 12)	−10.8 ± 3.0 mm	−11.1 ± 5.9 mm	0.4 ± 8.1 mm
After 2016 (N = 13)	−4.8 ± 1.6 mm	−6.6 ± 4.0 mm	1.7 ± 1.6 mm

N, number of stations in the sample.

**Table 5:** Mean differences and standard deviations in the up component between the compared solutions for the 25 stations analyzed.

Approach	E1–G1	E2–G1	E3–G2
DD (N = 25)	6.5 ± 6.1 mm	0.3 ± 4.1 mm	0.4 ± 3.0 mm
PPP (N = 25)	10.8 ± 5.6 mm	2.4 ± 5.1 mm	2.3 ± 4.8 mm

N, number of stations in the sample.

## Appendix A

**Table A1:** Coordinate differences between solution E1 and E2 (E2 minus E1) and their standard deviations. All values in millimeters.

Station	DD			PPP		
	dN	dE	dU	dN	dE	dU
NYA200NOR	10.2 ± 1.0	2.2 ± 1.1	−8.0 ± 3.7	9.7 ± 4.1	0.8 ± 3.7	−7.2 ± 52.1
OBE400DEU	9.6 ± 1.0	2.3 ± 0.9	−13.9 ± 3.5	8.5 ± 1.4	2.0 ± 3.4	−14.5 ± 7.5
BRUX00BEL	−0.4 ± 0.8	−0.2 ± 0.7	−14.0 ± 2.6	−1.1 ± 1.0	−0.2 ± 3.5	−15.2 ± 1.8
BADH00DEU	0.8 ± 1.1	−0.2 ± 0.9	−4.0 ± 3.4	0.4 ± 1.5	0.0 ± 4.0	−8.8 ± 5.9
GELL00DEU	−0.1 ± 1.0	0.0 ± 0.8	−8.3 ± 3.4	−1.0 ± 0.4	−0.5 ± 7.7	−8.8 ± 2.3
GOR200DEU	−0.6 ± 1.0	0.6 ± 0.9	−5.5 ± 3.3	−1.0 ± 1.0	−0.3 ± 3.0	−8.0 ± 6.5
WRLG00DEU	1.8 ± 0.7	1.9 ± 0.6	−14.8 ± 2.4	−0.3 ± 1.4	−0.3 ± 4.8	−13.0 ± 5.5
DOUR00BEL	0.4 ± 0.9	0.4 ± 0.8	−7.8 ± 3.0	−0.1 ± 0.4	0.2 ± 1.4	−9.2 ± 5.9
AUBG00DEU	0.4 ± 1.3	0.1 ± 1.1	−4.1 ± 4.1	−0.2 ± 6.0	−0.6 ± 8.8	−5.9 ± 7.7
BAUT00DEU	0.2 ± 1.1	0.0 ± 0.9	−3.5 ± 3.3	−0.4 ± 0.7	−0.6 ± 4.3	−5.5 ± 2.9
BORJ00DEU	0.1 ± 1.0	0.0 ± 0.9	−3.7 ± 3.1	−0.4 ± 1.6	−0.6 ± 2.3	−5.4 ± 3.4
DIEP00DEU	0.4 ± 0.9	−0.2 ± 0.8	−4.2 ± 3.3	−0.3 ± 0.5	−0.7 ± 1.5	−5.4 ± 2.1
DILL00DEU	0.6 ± 1.1	−0.1 ± 1.0	−4.5 ± 3.8	0.0 ± 1.5	−0.3 ± 2.2	−5.6 ± 7.1
EUSK00DEU	0.3 ± 1.0	−0.1 ± 0.8	−11.7 ± 3.0	−0.4 ± 0.8	−0.5 ± 2.2	−12.9 ± 3.9
HEL200DEU	1.0 ± 1.0	−0.1 ± 0.9	−4.3 ± 3.1	0.3 ± 1.0	−0.6 ± 2.8	−5.1 ± 4.3
HELG00DEU	0.0 ± 0.9	0.1 ± 0.8	−2.7 ± 2.8	−0.7 ± 0.9	−0.1 ± 2.1	−4.2 ± 2.8
HOFJ00DEU	0.7 ± 1.2	0.1 ± 1.1	−2.6 ± 3.8	0.1 ± 0.9	−0.4 ± 2.7	−4.7 ± 6.4
HOFN00ISL	1.2 ± 1.0	−0.1 ± 0.9	−11.1 ± 3.1	0.4 ± 1.0	−0.2 ± 1.6	−11.5 ± 1.3
ISTA00TUR	0.4 ± 1.1	0.1 ± 1.0	−5.8 ± 4.1	−0.2 ± 0.5	−0.4 ± 2.4	−9.1 ± 2.4
NICO00CYP	0.9 ± 1.0	−0.1 ± 0.9	−9.0 ± 3.4	0.1 ± 0.8	−0.2 ± 6.3	−12.8 ± 3.7
PTBB00DEU	1.0 ± 1.1	0.0 ± 0.9	−8.5 ± 3.3	0.6 ± 0.4	−0.6 ± 3.1	−9.7 ± 4.0
RANT00DEU	1.2 ± 1.0	0.0 ± 0.8	−3.5 ± 2.8	−0.1 ± 0.6	−0.6 ± 2.3	−4.7 ± 2.3
REYK00ISL	1.1 ± 1.2	−0.5 ± 1.1	−12.7 ± 3.8	0.2 ± 1.8	−0.4 ± 2.5	−13.2 ± 5.5
SAS200DEU	0.9 ± 1.1	−0.7 ± 0.9	−2.2 ± 3.4	0.1 ± 0.9	−0.4 ± 6.2	−5.0 ± 5.2
WARN00DEU	1.5 ± 1.0	−1.0 ± 0.8	−3.4 ± 3.0	−0.3 ± 0.6	0.2 ± 4.4	−4.6 ± 1.5
Mean	1.3 ± 2.6	0.2 ± 0.8	−7.0 ± 4.0	0.6 ± 2.6	−0.2 ± 0.6	−8.4 ± 3.5

**Table A2:** Coordinate differences between solution E1 and E3 (E3 minus E1) and their standard deviations. All values in millimeters.

Station	DD			PPP		
	dN	dE	dU	dN	dE	dU
NYA200NOR	9.8 ± 1.0	1.7 ± 1.1	−17.9 ± 3.7	10.3 ± 4.1	0.5 ± 3.7	−18.6 ± 52.4
OBE400DEU	10.2 ± 1.0	1.6 ± 0.9	−12.0 ± 3.4	10.1 ± 1.5	2.1 ± 3.5	−12.8 ± 7.8
BRUX00BEL	0.0 ± 0.8	−1.1 ± 1.1	−19.0 ± 2.6	−0.2 ± 1.0	−0.7 ± 3.5	−20.0 ± 1.8
BADH00DEU	−0.5 ± 1.1	−0.7 ± 0.9	−13.6 ± 3.4	−0.4 ± 1.5	−0.9 ± 4.2	−19.8 ± 5.9
GELL00DEU	−0.2 ± 0.9	−1.8 ± 0.8	−18.0 ± 3.4	−1.0 ± 0.5	−2.7 ± 7.3	−19.8 ± 2.3
GOR200DEU	−1.6 ± 1.0	0.2 ± 0.9	−8.1 ± 3.2	−1.9 ± 1.0	−1.2 ± 2.8	−10.7 ± 6.2
WRLG00DEU	1.0 ± 0.7	1.7 ± 0.6	−8.3 ± 2.4	−0.6 ± 1.4	−0.6 ± 4.7	−6.4 ± 5.6
DOUR00BEL	−1.0 ± 0.9	1.8 ± 0.8	−14.8 ± 3.0	−0.9 ± 0.5	1.2 ± 1.4	−16.1 ± 5.6
AUBG00DEU	−1.1 ± 1.3	−1.5 ± 1.1	−4.0 ± 4.1	−1.6 ± 6.0	−2.7 ± 8.8	−7.0 ± 7.3
BAUT00DEU	−1.0 ± 1.1	−2.1 ± 0.9	−5.3 ± 3.2	−1.5 ± 0.7	−3.0 ± 4.2	−7.7 ± 2.7
BORJ00DEU	−0.4 ± 1.0	1.4 ± 0.9	−2.5 ± 3.1	−0.7 ± 1.6	1.1 ± 2.2	−5.9 ± 3.7
DIEP00DEU	1.0 ± 0.9	−0.8 ± 0.8	−6.6 ± 3.3	0.4 ± 0.5	−1.8 ± 1.6	−6.6 ± 1.9
DILL00DEU	1.9 ± 1.1	−0.5 ± 1.0	−5.7 ± 3.8	1.5 ± 1.4	−1.3 ± 2.1	−7.3 ± 6.6
EUSK00DEU	0.9 ± 1.0	−0.4 ± 0.8	−10.1 ± 2.9	0.9 ± 0.8	−0.3 ± 2.1	−10.7 ± 3.9
HEL200DEU	0.7 ± 1.0	−0.9 ± 0.9	−3.9 ± 3.0	0.3 ± 1.0	−1.6 ± 2.7	−5.3 ± 4.6
HELG00DEU	−1.2 ± 0.9	−0.1 ± 0.8	−4.9 ± 2.8	−1.6 ± 0.9	−0.6 ± 1.9	−5.7 ± 3.2
HOFJ00DEU	1.2 ± 1.2	−0.6 ± 1.1	−2.5 ± 3.8	0.6 ± 0.9	−0.6 ± 2.8	−6.3 ± 6.0
HOFN00ISL	−1.1 ± 1.0	1.1 ± 0.9	−9.5 ± 3.1	−1.5 ± 0.9	0.9 ± 1.6	−9.9 ± 1.2
ISTA00TUR	1.4 ± 1.1	1.4 ± 1.0	0.1 ± 4.1	1.5 ± 0.6	1.1 ± 2.4	−3.3 ± 2.3
NICO00CYP	0.4 ± 1.0	0.0 ± 0.9	−6.6 ± 3.4	0.1 ± 0.8	0.0 ± 6.3	−10.3 ± 3.7
PTBB00DEU	−0.2 ± 1.1	−0.8 ± 0.9	0.1 ± 3.3	−0.4 ± 0.4	−0.9 ± 3.1	−2.3 ± 4.0
RANT00DEU	0.7 ± 1.0	−0.3 ± 0.8	−2.8 ± 2.8	−0.5 ± 0.6	−1.2 ± 2.1	−5.4 ± 2.2
REYK00ISL	0.4 ± 1.2	2.6 ± 1.1	−15.2 ± 3.8	0.0 ± 1.8	2.7 ± 2.5	−9.7 ± 5.4
SAS200DEU	0.2 ± 1.1	−0.1 ± 0.9	−4.2 ± 3.3	−0.6 ± 0.9	0.6 ± 6.0	−5.9 ± 4.9
WARN00DEU	0.2 ± 0.9	−2.5 ± 0.8	−4.7 ± 2.9	−2.2 ± 0.6	−0.8 ± 4.3	−5.6 ± 1.3
Mean	0.9 ± 2.8	0.0 ± 1.3	−8.0 ± 5.6	0.4 ± 3.1	−0.4 ± 1.4	−9.6 ± 5.2

**Table A3:** Coordinate differences between solution E1 and G1 (E1 minus G1) and their standard deviations. All values in millimeters.

Station	DD			PPP		
	dN	dE	dU	dN	dE	dU
NYA200NOR	−3.4 ± 1.0	5.9 ± 1.1	1.4 ± 3.9	−6.3 ± 2.9	9.0 ± 2.7	11.4 ± 35.5
OBE400DEU	−0.8 ± 1.1	2.2 ± 0.9	14.7 ± 3.4	3.0 ± 1.2	8.7 ± 2.8	16.9 ± 5.6
BRUX00BEL	−0.6 ± 0.8	1.0 ± 0.7	10.7 ± 2.5	1.5 ± 0.8	−0.7 ± 2.6	12.8 ± 2.2
BADH00DEU	0.0 ± 1.2	0.1 ± 1.0	7.3 ± 3.5	1.5 ± 1.4	−0.9 ± 3.1	14.0 ± 5.7
GELL00DEU	0.1 ± 1.0	−0.4 ± 0.8	17.9 ± 3.1	2.7 ± 0.4	3.4 ± 5.7	25.4 ± 2.1
GOR200DEU	−0.8 ± 1.1	−0.4 ± 0.9	4.6 ± 3.4	3.7 ± 1.1	−6.3 ± 2.3	11.8 ± 5.2
WRLG00DEU	0.3 ± 0.7	1.0 ± 0.6	−3.9 ± 2.4	3.8 ± 1.1	5.0 ± 3.5	9.3 ± 4.2
DOUR00BEL	1.6 ± 1.0	−0.9 ± 0.8	8.2 ± 3.1	4.0 ± 0.8	−5.8 ± 1.4	11.1 ± 4.7
AUBG00DEU	0.1 ± 1.3	0.3 ± 1.1	−7.1 ± 4.1	−2.9 ± 4.2	5.7 ± 6.1	−5.5 ± 5.7
BAUT00DEU	−0.8 ± 1.1	−0.1 ± 0.9	0.9 ± 3.4	3.4 ± 0.7	1.6 ± 3.3	8.2 ± 2.4
BORJ00DEU	−0.7 ± 1.1	0.6 ± 0.9	4.4 ± 3.2	1.9 ± 1.2	1.5 ± 1.7	6.3 ± 3.4
DIEP00DEU	0.0 ± 0.9	0.5 ± 0.8	1.3 ± 3.4	3.3 ± 0.4	1.6 ± 1.3	7.7 ± 2.8
DILL00DEU	0.4 ± 1.2	0.2 ± 1.0	4.4 ± 3.9	5.3 ± 1.1	6.0 ± 2.1	7.3 ± 5.3
EUSK00DEU	−0.6 ± 1.1	0.4 ± 0.9	13.0 ± 3.1	4.8 ± 0.7	6.3 ± 1.9	9.5 ± 3.5
HEL200DEU	−1.4 ± 1.1	0.3 ± 0.9	3.7 ± 3.2	1.6 ± 0.7	2.2 ± 2.0	6.5 ± 5.0
HELG00DEU	−0.1 ± 1.0	0.0 ± 0.8	5.4 ± 2.9	3.2 ± 1.1	−1.8 ± 1.9	11.2 ± 5.0
HOFJ00DEU	−2.6 ± 1.3	0.2 ± 1.1	2.8 ± 4.0	1.8 ± 0.8	−6.2 ± 2.1	11.5 ± 5.1
HOFN00ISL	0.0 ± 1.0	−1.5 ± 0.9	15.1 ± 3.2	−1.3 ± 0.8	2.9 ± 1.4	11.3 ± 2.1
ISTA00TUR	−0.9 ± 1.1	0.9 ± 1.0	13.0 ± 3.9	0.0 ± 0.6	2.0 ± 2.7	18.6 ± 1.9
NICO00CYP	−1.9 ± 1.0	−0.6 ± 1.0	7.3 ± 3.4	−2.2 ± 0.9	−0.2 ± 4.6	16.9 ± 2.9
PTBB00DEU	−1.0 ± 1.1	−0.1 ± 0.9	13.6 ± 3.3	3.1 ± 0.6	2.7 ± 2.2	16.5 ± 4.3
RANT00DEU	−0.6 ± 1.0	0.9 ± 0.8	1.4 ± 2.8	3.7 ± 1.0	2.3 ± 4.1	6.5 ± 2.5
REYK00ISL	−1.3 ± 1.3	−1.0 ± 1.2	13.6 ± 4.0	−2.2 ± 1.5	5.8 ± 2.6	9.9 ± 4.0
SAS200DEU	−1.5 ± 1.1	−0.3 ± 0.9	4.9 ± 3.3	4.1 ± 0.8	6.5 ± 4.5	9.0 ± 4.1
WARN00DEU	−0.7 ± 0.9	−0.4 ± 0.8	4.3 ± 2.9	3.9 ± 0.6	0.7 ± 3.2	5.1 ± 3.1
Mean	−0.7 ± 1.0	0.4 ± 1.4	6.5 ± 6.1	1.8 ± 2.8	2.1 ± 4.1	10.8 ± 5.6

**Table A4:** Coordinate differences between solution E2 and G1 (E2 minus G1) and their standard deviations. All values in millimeters.

Station	DD			PPP		
	dN	dE	dU	dN	dE	dU
NYA200NOR	6.8 ± 1.0	8.1 ± 1.1	−6.5 ± 3.9	3.5 ± 3.0	9.8 ± 2.7	4.2 ± 38.3
OBE400DEU	8.7 ± 1.1	4.5 ± 0.9	0.8 ± 3.5	11.5 ± 1.2	10.7 ± 2.8	2.5 ± 5.2
BRUX00BEL	−1.0 ± 0.8	0.8 ± 0.7	−3.5 ± 2.6	0.4 ± 0.8	−1.0 ± 2.7	−2.4 ± 2.1
BADH00DEU	0.8 ± 1.2	−0.1 ± 1.0	3.3 ± 3.5	1.9 ± 1.2	−0.9 ± 2.9	5.2 ± 3.1
GELL00DEU	0.0 ± 1.0	−0.5 ± 0.8	9.4 ± 3.1	1.7 ± 0.4	2.9 ± 5.4	16.6 ± 2.1
GOR200DEU	−1.4 ± 1.1	0.2 ± 0.9	−0.8 ± 3.4	2.8 ± 1.1	−6.6 ± 2.1	3.8 ± 5.0
WRLG00DEU	0.5 ± 0.7	0.7 ± 0.6	0.9 ± 2.4	3.6 ± 1.1	4.7 ± 3.4	−3.6 ± 4.0
DOUR00BEL	2.0 ± 1.0	−0.4 ± 0.8	0.4 ± 3.1	3.9 ± 0.8	−5.5 ± 1.4	1.9 ± 4.5
AUBG00DEU	0.6 ± 1.3	0.4 ± 1.1	−11.2 ± 4.1	−3.1 ± 4.3	5.2 ± 6.3	−11.4 ± 5.6
BAUT00DEU	−0.6 ± 1.1	−0.1 ± 0.9	−2.6 ± 3.4	2.9 ± 0.6	1.0 ± 3.2	2.7 ± 2.4
BORJ00DEU	−0.6 ± 1.1	0.5 ± 0.9	0.8 ± 3.2	1.4 ± 1.2	0.9 ± 1.6	1.0 ± 3.4
DIEP00DEU	0.4 ± 1.0	0.3 ± 0.8	−2.9 ± 3.4	3.0 ± 0.4	0.9 ± 1.3	2.2 ± 2.7
DILL00DEU	1.0 ± 1.2	0.1 ± 1.0	−0.1 ± 3.9	5.3 ± 1.1	5.7 ± 2.1	1.7 ± 5.1
EUSK00DEU	−0.3 ± 1.1	0.3 ± 0.9	1.2 ± 3.1	4.4 ± 0.6	5.8 ± 1.9	−3.4 ± 3.4
HEL200DEU	−0.4 ± 1.1	0.2 ± 0.9	−0.6 ± 3.2	2.0 ± 0.8	1.5 ± 2.1	1.4 ± 5.1
HELG00DEU	−0.2 ± 1.0	0.0 ± 0.8	2.7 ± 2.9	2.5 ± 1.1	−1.9 ± 1.9	7.0 ± 4.9
HOFJ00DEU	−1.9 ± 1.3	0.3 ± 1.1	0.3 ± 4.0	1.9 ± 0.8	−6.7 ± 2.2	6.8 ± 5.0
HOFN00ISL	1.2 ± 1.0	−1.6 ± 0.9	4.1 ± 3.2	−0.9 ± 0.8	2.7 ± 1.3	−0.2 ± 2.1
ISTA00TUR	−0.5 ± 1.1	1.1 ± 1.0	7.2 ± 3.9	−0.1 ± 0.6	1.7 ± 2.2	9.6 ± 1.9
NICO00CYP	−1.0 ± 1.0	−0.7 ± 1.0	−1.7 ± 3.4	−2.0 ± 0.9	−0.4 ± 4.6	4.1 ± 2.9
PTBB00DEU	0.0 ± 1.1	0.0 ± 0.9	5.0 ± 3.3	3.7 ± 0.6	2.1 ± 2.2	6.8 ± 4.3
RANT00DEU	0.6 ± 1.0	0.9 ± 0.8	−2.2 ± 2.8	3.7 ± 0.5	1.7 ± 1.7	1.9 ± 2.4
REYK00ISL	−0.2 ± 1.3	−1.6 ± 1.2	1.0 ± 4.0	−2.0 ± 1.5	5.4 ± 2.6	−3.3 ± 4.0
SAS200DEU	−0.5 ± 1.1	0.1 ± 1.0	2.7 ± 3.5	4.2 ± 0.8	6.1 ± 4.4	4.1 ± 4.0
WARN00DEU	0.2 ± 1.0	−0.2 ± 0.8	0.8 ± 3.0	3.5 ± 0.6	0.9 ± 3.2	0.5 ± 3.1
Mean	0.6 ± 2.3	0.5 ± 1.9	0.3 ± 4.1	2.4 ± 2.8	1.9 ± 4.3	2.4 ± 3.1

**Table A5:** Coordinate differences between solution E3 and G2 (G2 minus E3) and their standard deviations. All values in millimeters.

Station	DD			PPP		
	dN	dE	dU	dN	dE	dU
NYA200NOR	6.7 ± 1.0	8.3 ± 1.1	−1.7 ± 3.9	3.7 ± 2.9	9.5 ± 2.6	7.3 ± 38.6
OBE400DEU	8.3 ± 1.1	5.0 ± 0.9	0.6 ± 3.5	12.2 ± 1.2	11.2 ± 2.8	3.1 ± 5.6
BRUX00BEL	−1.7 ± 0.8	1.1 ± 0.7	−2.7 ± 2.6	0.0 ± 0.8	−1.1 ± 2.7	−2.0 ± 2.1
BADH00DEU	−0.1 ± 1.2	0.7 ± 0.9	7.1 ± 3.4	1.6 ± 1.2	0.9 ± 3.1	8.9 ± 3.0
GELL00DEU	−0.3 ± 0.9	0.2 ± 0.8	3.5 ± 3.1	0.7 ± 0.4	3.5 ± 4.8	11.2 ± 2.1
GOR200DEU	−1.6 ± 1.1	0.6 ± 0.9	−1.1 ± 3.4	2.0 ± 1.0	−6.3 ± 1.8	4.6 ± 4.6
WRLG00DEU	0.3 ± 0.7	1.1 ± 0.6	−0.5 ± 2.4	3.2 ± 1.1	4.5 ± 3.3	−4.5 ± 4.1
DOUR00BEL	1.7 ± 1.0	−0.2 ± 0.8	1.3 ± 3.1	3.6 ± 0.8	−4.9 ± 1.4	3.4 ± 4.1
AUBG00DEU	−0.1 ± 1.3	1.2 ± 1.1	−9.6 ± 4.1	−4.3 ± 4.2	5.6 ± 6.4	−10.9 ± 5.1
BAUT00DEU	−1.0 ± 1.1	0.5 ± 0.9	−0.9 ± 3.3	1.8 ± 0.6	1.1 ± 3.0	3.1 ± 2.1
BORJ00DEU	−0.9 ± 1.1	0.6 ± 0.9	0.7 ± 3.2	0.7 ± 1.1	1.0 ± 1.5	0.6 ± 3.7
DIEP00DEU	−0.1 ± 0.9	0.5 ± 0.8	−0.7 ± 3.3	2.1 ± 0.3	1.7 ± 1.3	2.9 ± 2.6
DILL00DEU	0.0 ± 1.2	0.8 ± 1.0	−0.3 ± 3.8	3.8 ± 1.0	6.8 ± 2.0	1.9 ± 4.4
EUSK00DEU	−0.7 ± 1.1	0.6 ± 0.9	1.1 ± 3.1	4.3 ± 0.7	5.9 ± 1.8	−3.7 ± 3.4
HEL200DEU	−0.5 ± 1.1	0.7 ± 0.9	−1.0 ± 3.2	1.5 ± 0.8	2.1 ± 1.9	0.5 ± 5.4
HELG00DEU	−0.4 ± 1.0	0.5 ± 0.8	2.9 ± 2.9	1.8 ± 1.1	−1.8 ± 1.7	7.5 ± 5.2
HOFJ00DEU	−2.5 ± 1.3	0.8 ± 1.1	1.4 ± 3.9	0.3 ± 0.8	−6.9 ± 2.3	7.5 ± 4.5
HOFN00ISL	1.7 ± 1.0	−1.5 ± 0.9	2.9 ± 3.2	−0.7 ± 0.8	2.8 ± 1.3	−0.5 ± 2.0
ISTA00TUR	−1.2 ± 1.1	1.6 ± 1.0	4.0 ± 3.9	−0.2 ± 0.7	0.6 ± 2.2	5.8 ± 1.8
NICO00CYP	−1.8 ± 1.0	−0.2 ± 1.0	−1.1 ± 3.5	−2.0 ± 0.8	−0.2 ± 4.6	4.1 ± 2.8
PTBB00DEU	0.1 ± 1.1	0.3 ± 0.9	2.7 ± 3.3	3.5 ± 0.6	2.2 ± 2.2	3.7 ± 4.3
RANT00DEU	0.8 ± 0.9	1.2 ± 0.8	−2.5 ± 2.8	3.0 ± 0.5	2.5 ± 1.5	1.5 ± 2.4
REYK00ISL	−0.1 ± 1.3	−2.0 ± 1.2	−0.4 ± 4.0	−2.5 ± 1.5	4.9 ± 2.6	−4.4 ± 3.9
SAS200DEU	−1.1 ± 1.1	0.2 ± 0.9	1.8 ± 3.3	3.1 ± 0.7	6.3 ± 4.1	4.3 ± 3.7
WARN00DEU	−0.2 ± 0.9	0.4 ± 0.8	1.3 ± 2.9	2.9 ± 0.6	0.5 ± 3.1	1.1 ± 3.1
Mean	0.2 ± 2.4	0.9 ± 1.9	0.4 ± 3.0	1.9 ± 3.0	2.1 ± 4.3	1.3 ± 4.8

**Table A6:** Coordinate differences between solution G1 and G2 (G2 minus G1) and their standard deviations. All values in millimeters.

Station	DD			PPP		
	dN	dE	dU	dN	dE	dU
NYA200NOR	0.3 ± 1.1	0.7 ± 1.1	14.7 ± 4.1	−0.3 ± 0.4	0.0 ± 0.7	14.5 ± 1.7
OBE400DEU	−1.0 ± 1.1	1.2 ± 1.0	−2.1 ± 3.5	−0.9 ± 0.8	0.4 ± 1.9	−1.0 ± 1.5
BRUX00BEL	1.2 ± 0.8	1.2 ± 0.7	5.8 ± 2.5	−1.3 ± 0.5	0.4 ± 1.3	5.2 ± 2.5
BADH00DEU	0.4 ± 1.2	1.3 ± 1.0	13.5 ± 3.5	0.5 ± 1.0	2.7 ± 1.4	14.6 ± 2.7
GELL00DEU	−0.1 ± 1.0	2.5 ± 0.8	3.8 ± 2.8	−1.0 ± 0.3	2.8 ± 1.5	5.6 ± 1.8
GOR200DEU	0.9 ± 1.1	0.8 ± 1.0	2.3 ± 3.5	0.2 ± 1.1	1.2 ± 0.8	3.5 ± 3.2
WRLG00DEU	0.7 ± 0.8	0.6 ± 0.7	−7.8 ± 2.4	0.0 ± 0.6	0.1 ± 0.8	−7.4 ± 1.8
DOUR00BEL	1.1 ± 1.0	−1.2 ± 0.8	7.9 ± 3.2	0.5 ± 1.1	−0.4 ± 1.4	8.4 ± 2.6
AUBG00DEU	0.8 ± 1.4	2.5 ± 1.2	1.5 ± 4.1	0.2 ± 0.6	2.6 ± 1.0	1.7 ± 2.4
BAUT00DEU	0.8 ± 1.1	2.7 ± 1.0	3.5 ± 3.5	0.0 ± 0.5	2.4 ± 1.5	2.6 ± 1.7
BORJ00DEU	0.2 ± 1.2	−1.4 ± 1.0	−1.3 ± 3.4	−0.4 ± 0.4	−1.6 ± 0.5	0.2 ± 3.4
DIEP00DEU	−1.1 ± 1.0	0.8 ± 0.8	4.6 ± 3.4	−1.6 ± 0.3	1.9 ± 1.0	1.8 ± 3.3
DILL00DEU	−2.3 ± 1.3	1.2 ± 1.1	1.0 ± 3.9	−3.0 ± 0.5	2.2 ± 1.9	1.9 ± 2.0
EUSK00DEU	−0.1 ± 1.2	0.7 ± 1.0	−1.7 ± 3.3	−1.4 ± 0.4	−0.1 ± 1.5	−2.5 ± 3.0
HEL200DEU	0.2 ± 1.1	1.3 ± 0.9	−0.7 ± 3.3	−0.5 ± 0.4	1.6 ± 0.6	−0.7 ± 5.8
HELG00DEU	0.9 ± 1.1	0.6 ± 0.9	2.4 ± 3.1	0.2 ± 1.3	0.6 ± 1.7	2.0 ± 6.4
HOFJ00DEU	−1.1 ± 1.4	1.1 ± 1.2	1.0 ± 4.1	−2.0 ± 0.6	−0.1 ± 1.5	2.2 ± 3.2
HOFN00ISL	2.8 ± 1.1	−1.1 ± 0.9	−2.8 ± 3.3	2.1 ± 0.6	−0.9 ± 1.1	−1.9 ± 2.6
ISTA00TUR	−1.7 ± 1.1	−0.7 ± 1.1	−9.1 ± 3.6	−1.7 ± 0.7	−2.6 ± 2.0	−9.6 ± 1.4
NICO00CYP	−0.3 ± 1.1	0.4 ± 1.1	−1.8 ± 3.5	0.1 ± 0.9	0.0 ± 1.4	−2.5 ± 1.7
PTBB00DEU	1.4 ± 1.1	1.1 ± 0.9	−11.0 ± 3.3	0.8 ± 0.8	0.3 ± 0.5	−10.5 ± 4.7
RANT00DEU	0.7 ± 0.9	0.6 ± 0.8	−1.0 ± 2.8	−0.2 ± 0.4	1.4 ± 0.9	0.4 ± 2.6
REYK00ISL	0.7 ± 1.4	−3.5 ± 1.2	1.2 ± 4.1	−0.3 ± 1.1	−3.7 ± 2.7	−4.6 ± 1.3
SAS200DEU	0.1 ± 1.1	−0.6 ± 0.9	1.1 ± 3.4	−0.4 ± 0.6	−0.8 ± 1.3	1.2 ± 2.4
WARN00DEU	0.9 ± 1.0	2.1 ± 0.8	1.8 ± 2.9	1.3 ± 0.6	0.5 ± 1.0	1.5 ± 4.2
Mean	0.3 ± 1.1	0.6 ± 1.4	1.1 ± 5.7	−0.4 ± 1.1	0.4 ± 1.6	1.1 ± 5.9
